# Small airways morphological alterations associated with functional impairment in lymphangioleiomyomatosis

**DOI:** 10.1186/s12890-023-02837-2

**Published:** 2024-01-09

**Authors:** Lígia Pelosi Mendonça, Natalia de Souza Xavier Costa, Ellen Caroline Toledo do Nascimento, Martina Rodrigues de Oliveira, Carlos Roberto Ribeiro de Carvalho, Bruno Guedes Baldi, Marisa Dolhnikoff

**Affiliations:** 1 Departamento de Patologia, Faculdade de Medicina, Universidade de Sao Paulo, Av. Dr. Arnaldo, 455, Cerqueira Cesar, CEP 01246-903 Sao Paulo, SP Brasil; 2 Divisao de Pneumologia, Instituto do Coracao (InCor), Faculdade de Medicina, Hospital das Clinicas HCFMUSP, Universidade de Sao Paulo, Sao Paulo, SP Brasil

**Keywords:** Lymphangioleiomyomatosis, Small airways, Pulmonary function test, LAM Score, Immunohistochemistry

## Abstract

**Background:**

Lymphangioleiomyomatosis (LAM) is a rare neoplastic and cystic pulmonary disease characterized by abnormal proliferation of the so-called LAM cells. Despite the functional obstructive pattern observed in most patients, few studies investigated the morphological changes in the small airways, most of them in patients with severe and advanced LAM undergoing lung transplantation. Understanding the morphological changes in the airways that may occur early in the disease can help us understand the pathophysiology of disease progression and understand the rationale for possible therapeutic approaches, such as the use of bronchodilators. Our study aimed to characterize the morphological alterations of the small airways in patients with LAM with different severities compared to controls, and their association with variables at the pulmonary function test and with LAM Histological Score (LHS).

**Methods:**

Thirty-nine women with LAM who had undergone open lung biopsy or lung transplantation, and nine controls were evaluated. The histological severity of the disease was assessed as LHS, based on the percentage of tissue involvement by cysts and infiltration by LAM cells. The following morphometric parameters were obtained: airway thickness, airway closure index, collagen and airway smooth muscle content, airway epithelial TGF-β expression, and infiltration of LAM cells and inflammatory cells within the small airway walls.

**Results:**

The age of patients with LAM was 39 ± 8 years, with FEV1 and DLCO of 62 ± 30% predicted and 62 ± 32% predicted, respectively. Patients with LAM had increased small airway closure index, collagen and smooth muscle content, and epithelial TGF-beta expression compared with controls. Patients with LAM with the more severe LHS and with greater functional severity (FEV1 ≤ 30%) presented higher thicknesses of the airways. Bronchiolar inflammation was mild; infiltration of the small airway walls by LAM cells was rare. LHS was associated with an obstructive pattern, air trapping, and reduced DLCO, whereas small airway wall thickness was associated with FEV1, FVC, and collagen content.

**Conclusion:**

LAM is associated with small airway remodelling and partial airway closure, with structural alterations observed at different airway compartments. Functional impairment in LAM is associated with airway remodelling and, most importantly, with histological severity (LHS).

## Background

Lymphangioleiomyomatosis (LAM) is a rare systemic disease characterized by the occurrence of diffuse cysts in the lung parenchyma, predominantly in women at reproductive years [1]. The etiology of LAM is still unclear, and it can occur sporadically (sLAM) or be related to the Tuberous Sclerosis Complex (TSC-LAM). The sporadic form is responsible for most cases, with an estimated prevalence of approximately 3.3 to 7.7 per 1,000,000 women [2, 3]. Tuberous sclerosis is a dominant autosomal disease, associated with the suppressor oncogenes TSC-1 in chromosome 9, and TSC-2 in chromosome 16, and mutations in their alleles may lead to cellular proliferation [4]. It is estimated that about 80% of women with TSC will present pulmonary cysts in the follow-up [5].

The diagnosis of LAM is often obtained when the patient is between 30 and 40 years old, but clinical manifestations are often observed months or years before the diagnosis [6, 7]. The main clinical manifestations include pneumothorax, and dyspnea [8]. The disease is characterized by abnormal proliferation of the so-called LAM cells: atypical muscle-like cells that resemble smooth muscle in their histological and ultrastructural constitution, located in the pulmonary peribronchial and perivascular interstitium and in thoracic and abdominal lymphatics, which infiltrate the lung as LAM nodules and are typically associated with cystic lung destruction [9–11]. Due to its metastatic properties, LAM is considered as a low-grade neoplastic disease with a transplant-free survival of 10 years for 86% of the cases [11–13].

The most frequent abnormalities on the pulmonary functions tests (PFT) are reduced diffusion capacity for carbon monoxide (DLCO), air trapping, and airflow obstruction, which may not be present early in the disease but becomes evident as the disease progresses [14, 15]. Previous studies have determined how lung cystic destruction and variability in LAM nodules biology are associated with clinical features and loss of pulmonary function [16–19].

Despite the functional obstructive pattern observed in most patients with LAM, few studies to date have investigated the morphological changes in the small airways that may be associated with the disease. In a report of three patients that died of LAM, Sobonya et al. (1985) [18] reported a significant decrease of small airway diameters, with similar amounts of smooth muscle when compared with controls. Small airways appeared to be collapsed, suggesting a loss of support by alveolar parenchyma with “emphysema-like lesions”. The authors did not correlate morphological findings with lung function data but suggested that the airspace lesions of LAM were more important in the genesis of expiratory airflow limitation than were the occasional small airways that appeared to contain an excess of smooth muscle. Taveira DaSilva et al. (2001) [19] observed airway inflammation in 47/74 (63%) lung biopsies of patients with LAM, and airway smooth muscle hyperplasia in few (4%) cases. These authors showed that LAM patients who responded more frequently to bronchodilators in the PFT had a predominantly solid pattern of LAM lesion surrounding the airways, but airway inflammation was not a predictor of a positive response. Hayashi et al. (2016) [20] reported irregular proliferation of lymphatic vessels in the airways and changes associated with chronic inflammation, in addition to the proliferation of LAM cells around the bronchi in patients with advanced LAM who underwent lung transplantation. More recently, Verleden et al. (2020) [21] studied explant lungs from patients undergoing lung transplantation for end-stage LAM using a combination of ex vivo computed tomography (CT), microCT, and histology. The authors reported a significant decrease in the number of small airways in severe-LAM patients, caused by cystic destruction, as well as thickening of terminal bronchioles, suggesting that these small airway alterations would contribute to the progressive loss of lung function. To date, no study investigated the small airway changes in LAM at earlier stages of the disease. In the present study, we aimed to characterize the morphological alterations of the small airways in patients with LAM with different functional severities, who underwent surgical lung biopsy for diagnostic purposes or lung transplantation, and their association with parameters obtained in the PFTs and with a morphological parameter of disease severity (LAM histological score).

## Materials and methods

This study was approved by the review board for the human ethics committee of the University of Sao Paulo (CAPPesq-FMUSP; CAAE: 68159817.1.0000.0068).

### Study Population

We included 39 adult women with a clinical-radiological diagnosis and histopathological confirmation of LAM who had undergone surgical lung biopsy (n = 27) or lung transplantation (n = 12) between 1994 and 2016. Clinical and functional variables were assessed in the medical charts.

Nine subjects that died from non-pulmonary diseases and with normal lung histology were selected as controls and assessed during routine autopsies. Controls were non-smokers, did not have any chronic lung disease, and had no need for mechanical ventilation. Informed consent was obtained from all subjects and/or their legal guardians. Autopsies were requested by the clinical staff and were performed at the Death Verification Service (SVOC), an autopsy service linked to the Clinical Hospital at Sao Paulo University.

### Pulmonary function tests

Spirometry, lung volumes, and DLCO were performed by using a calibrated pneumotachograph and a body plethysmograph, respectively (Medical Graphics Corporation and Elite Dx, Elite Series; Medical Graphics Corporation), and according to recommended standards [22]. Forced vital capacity (FVC), forced expiratory volume in the first second (FEV1), FEV1/FVC, total lung capacity (TLC), residual volume (RV), RV/TLC, and DLCO were obtained, and predicted values for the Brazilian population were used [22–25]. Functional variables were evaluated within one year before lung biopsy or lung transplantation.

According to functional parameters, we subdivided the patients with LAM into two groups based on disease severity: (1) predicted FEV1 ≥ 70%: the less severe group, and (2) predicted FEV1 ≤ 30%: the more severe group.

### LAM histological severity score (LHS)

Surgical lung biopsies and fragments of peripheral tissue of lung explants were fixed in buffered 10% formalin and routinely processed. Tissue sections were stained with hematoxylin and eosin (H&E) for routine analysis and with Sirius Red for collagen detection. Immunohistochemistry (IH) was performed using anti-smooth muscle alpha-actin (SMA), anti-HMB45, and anti-TGF-β antibodies. The slides were scanned using the Pannoramic Flash Scanner (3D Histech, Budapest, Hungary).

The histological severity of the disease was assessed as LAM Histologic Score (LHS) in the slides immunostained with SMA. LHS was previously developed and has been used to grade the histological severity of disease [26]. The grading system is based on the percentage of tissue involvement by cysts and infiltration by LAM cells and has been associated with clinical severity and loss of lung function [14, 27, 28]. LAM lesions were graded in scores 1 to 3, according to the total percentage of lung tissue involvement by both the proliferative and the cystic components of the lesions, as follows: LHS-1, < 25%; LHS-2, 25–50%; and LHS-3, > 50% of lung involvement [26]. According to the LHS, we further subdivided the LAM patients into two groups of histological severity: Less Severe (LHS-1 + LHS-2), corresponding to lung involvement up to 50%, and More Severe, corresponding to LHS-3, > 50% of lung tissue involvement.

### Morphometrical analysis

Transversally cut small airways [with basement membrane (BM) perimeter ≤ 6 mm] with preserved architecture were selected in Sirius Red stained slides from LAM and control individuals. Transverse sections are those with a short/long diameter ratio greater than 0.6 and with even thickness of airway epithelium and inner airway wall [29, 30], meaning that a perfectly transversely cut airway looks like a circle and has a short/long diameter ratio ≅ 1.0. The preserved airway architecture refers to preserved morphological structure, with well-defined airway layers (epithelium, basement membrane, smooth muscle and adventitia), that is, airways that are not yet distorted and involved by LAM cysts. To avoid selection bias, all transversally cut airways were measured, varying from 4 to 6 airways in each individual. Airways involved by the cystic destruction of lung parenchyma were not included in the analysis. Images were analyzed using Image-Pro Plus 4.1 software (Media Cybernetics, Silver Spring, MD, USA). The following morphological parameters were obtained: (1) airway wall thickness (um) - corresponds to airway wall area (um^2^) divided by the corresponding BM perimeter (um); (2) airway closure index (%) - is a measurement that reflects the decrease in lumen area, whether due to airway constriction or thickening, when compared to the airway lumen in its relaxed state. It corresponds to the difference between the measured airway lumen area and the predicted relaxed lumen area, calculated as follows: [1- (measured lumen area (um²) / predicted relaxed lumen area (um²))] x 100. The predicted area of the relaxed airway lumen was calculated as πR^2^, where R = airway lumen radius. The lumen area was delimited from the BM [31]; (3) collagen content - corresponds to the area of airway wall collagen divided by the perimeter of BM (um²/um); (4) airway smooth muscle content - corresponds to the area of airway smooth muscle divided by the BM perimeter (um²/um); and (5) airway epithelial TGF-β expression (assessed in TGF-β immunostained slides) - corresponds to the area of immunopositive TGF-β epithelial cells divided by the BM perimeter (um²/um).

We also evaluated the presence of infiltration of LAM cells within small airway walls (HMB-45 immunostained slides) and the presence of bronchiolar inflammatory cell infiltration (H&E-stained slides).

### Statistical analysis

SPSS 20 software (SPSS Inc/IBM, Chicago, USA) was used for the statistical analysis. Data distribution was assessed by the Kolmogorov-Smirnov normality test followed by T-student or Mann-Whitney test. Statistical difference was assumed at the 5% significance level. For each individual, mean values of each histological variable were calculated. Data are presented as mean ± standard deviation or as numbers and proportions.

Correlation tests were performed using RStudio, version 4.1.1 (RStudio, PBC, Boston, MA, USA) adopting a 5% significance level. P-values were corrected for multiple comparisons by the Benjamini-Hochberg method.

## Results

Thirty-nine patients with LAM were included in the study. One patient did not perform pulmonary function tests (n = 38). IH for α-SMA and TGF-β could not be performed in one of the biopsies due to the scarcity of tissue in the paraffin block (n = 38).

Table 1 shows the clinical and functional data of patients with LAM and LAM histological scores. The age of patients with LAM was 39.5 ± 8.2 years. Thirty-five patients (89.7%) had sLAM and 4 (10.3%) had TSC-LAM.

The age of the control patients was 50.7 ± 10.3 years. The most common causes of death in the control group were sudden cardiovascular death, including acute myocardial infarction, heart failure, cardiogenic shock, and cardiogenic pulmonary edema.

**Table 1:** Clinical, functional and LHS data of patients with LAM

Clinical data	N	LAM
Age	39	39.5 ± 8.2
Smoking status- n (%)	39	
- Former-smokers - Current smokers		6 (15.4) 1 (2.5)
Dyspnea - n (%)	39	18 (46.1)
Pneumothorax - n (%)	39	16 (41.0)
Angiomyolipoma - n (%)	39	11 (28.2)
Tuberous Sclerosis - n (%)	39	4 (10.3)
Treatment at the time of surgery - n (%)		
- Sirolimus - Inhaled corticosteroids - Long-acting bronchodilator		2 (5.1) 2 (5.1) 6 (15.4)
**Pulmonary function test**		
FEV1 (L)	38	1.8 ± 0.8
FEV1 (% predicted)	38	62.4 ± 30.0
FVC (L)	38	2.8 ± 0.8
FVC (% predicted)	37	80.7 ± 24.7
FEV1/FVC	38	0.6 ± 0.2
DLCO (mL/min/mmHg)	33	16.2 ± 8.2
DLCO (% predicted)	34	62.5 ± 31.8
TLC (L)	33	5.2 ± 1.1
TLC (% predicted)	34	105.4 ± 17.7
RV (L)	33	2.2 ± 1.0
RV (% predicted)	34	153.7 ± 59.1
RV/TLC	33	0.4 ± 0.1
**LAM histological score (LHS)**		
Up to 50%	38	22 (57.9%)
Over 50%	38	16 (42.1%)

Data are presented as mean ± standard deviation. FEV1: forced expiratory volume in the first second, FVC: forced vital capacity, DLCO: lung diffusion capacity for carbon monoxide, TLC: total lung capacity, RV: residual volume, L: liters, % predicted: percentage of predicted value

**Fig. 1:**
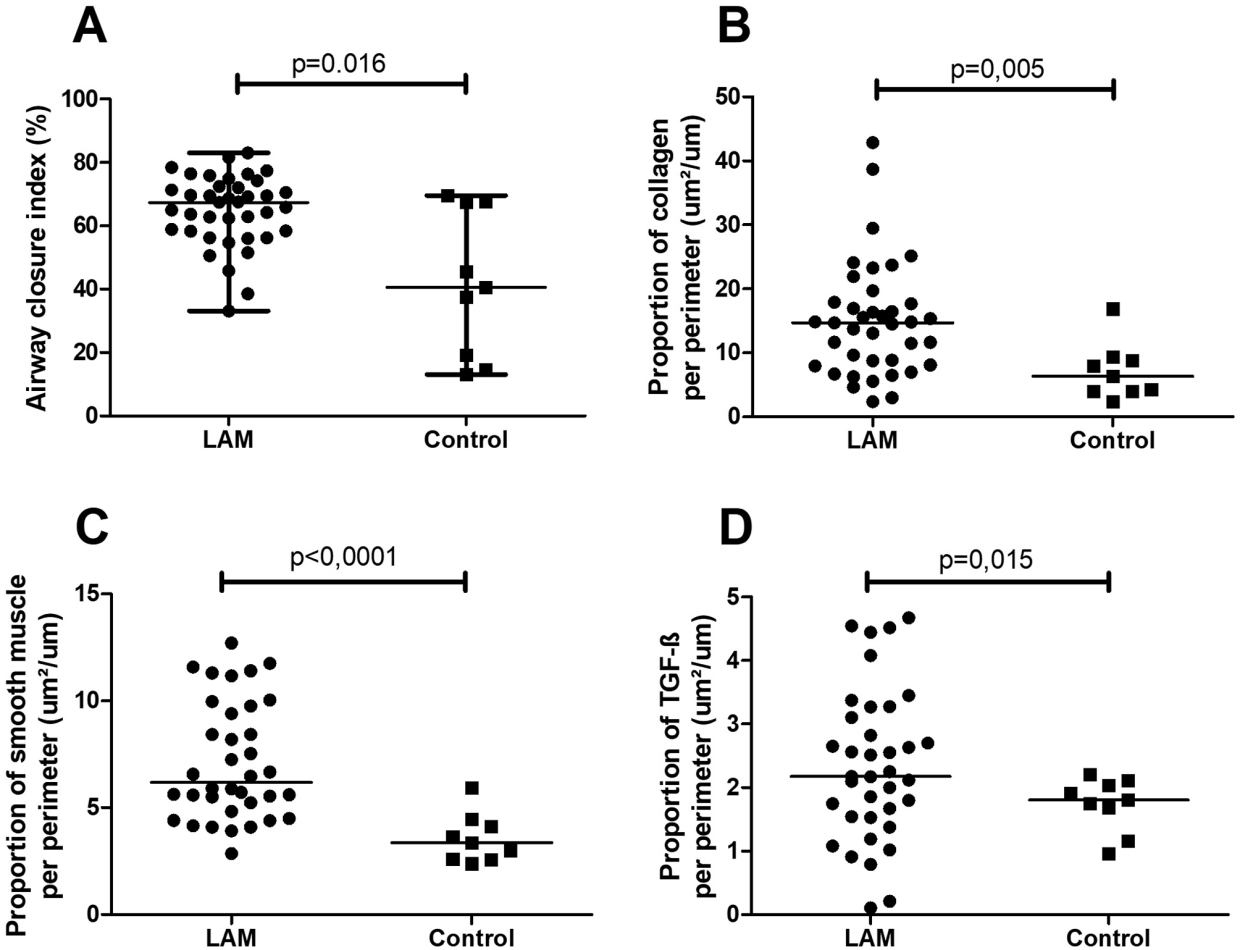
Graphical representation of (**A**) small airway closure index; (**B**) airway collagen content; (**C**) airway smooth muscle content; and (**D**) airway epithelial TGF-β expression. Each data point represents one individual. Bars represent mean values.

The LAM group presented increased small airway closure index (p = 0.016), collagen content (p = 0.005), smooth muscle content (p ≤ 0.0001), and epithelial TGF-beta expression (p = 0.015) compared with controls (Fig. 1). There was no statistical difference in the airway thickness between the two groups.

**Fig. 2:**
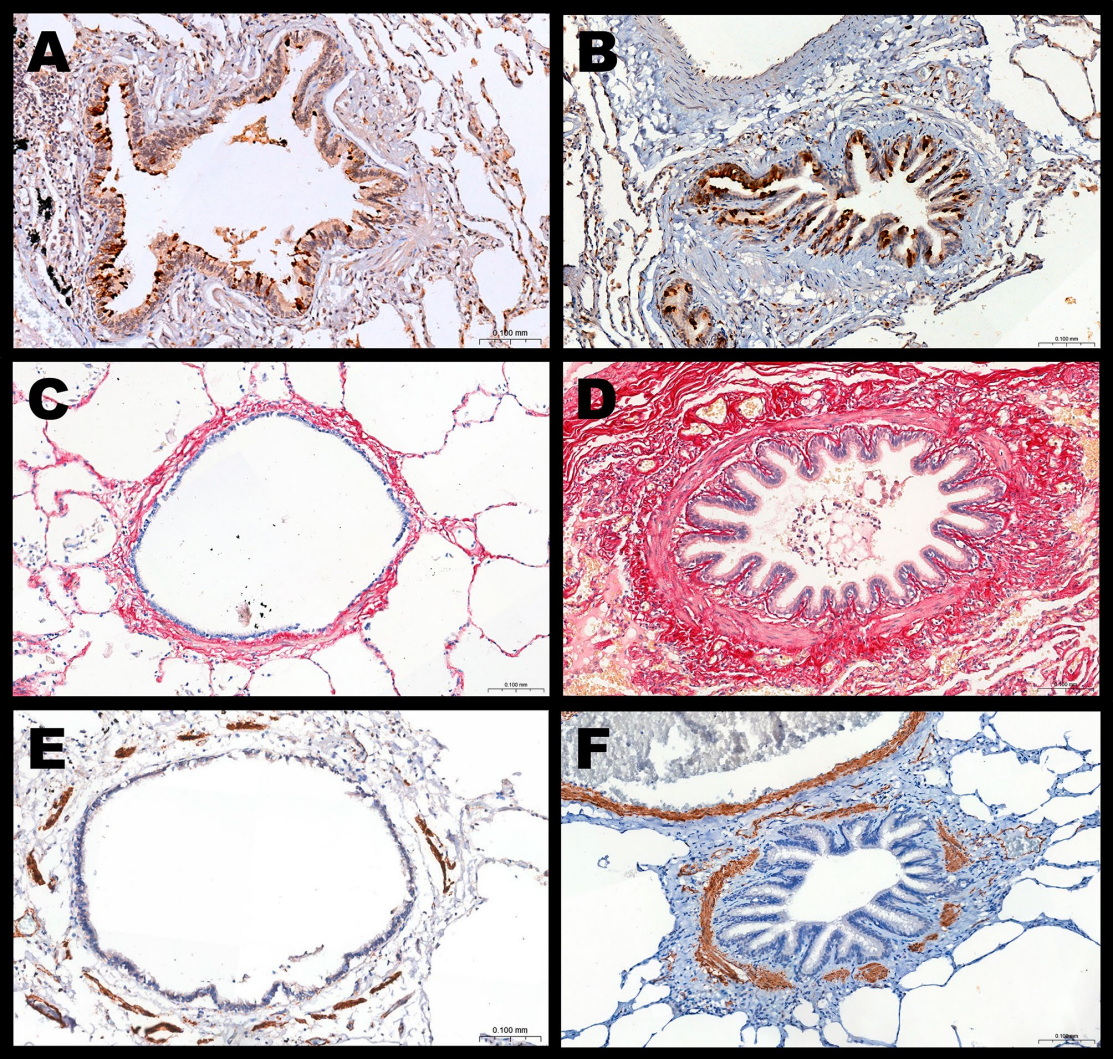
Representative photomicrographs of small airways in the control group (**A**, **C**, **E**) and patients with LAM (**B**, **D**, **F**). **A and B**: Epithelial expression of TGF-β; **C and D**: Collagen content at Sirius Red staining. **E and F**: Smooth muscle content in SMA immunostaining.

Figure 2 shows representative photomicrographs of the main observed morphological differences between patients with LAM and controls. Increased epithelial TGF-β, collagen content, airway smooth muscle (ASM) content, and partial airway closure can be observed in patients with LAM.

**Fig. 3:**
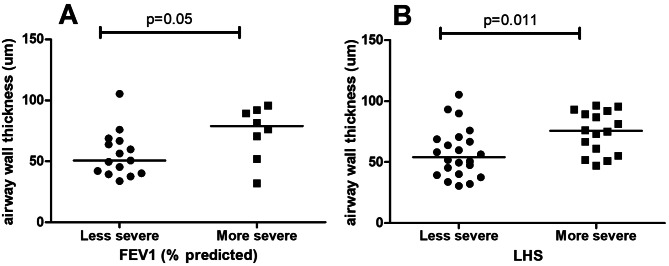
Graphical representation of the airway thickness. Comparison between the most and least severe cases of LAM from a functional point of view (**A**), and according to the histological score (**B**). Each data point represents one individual. Bars represent mean values.

When comparing the LAM group divided according to the histological severity [LHS up to 50% (n = 22) vs. LHS over 50% (n = 16)] and functional severity [FEV1 ≥ 70% (n = 15) vs. FEV1 ≤ 30% (n = 8)], airway thickness was the only morphological variable with significant difference (Fig. 3). LAM cases with FEV1 ≤ 30% (more severe) showed a tendency towards higher airway thickness (73.7 ± 21.9) compared to those with FEV1 ≥ 70% (less severe) (55.7 ± 18.8; p = 0.05) (Fig. 3A). In addition, patients with LAM with the more severe LHS presented higher thicknesses of the airways compared to those with less severe LHS (p = 0.011) (Fig. 3B).

**Fig. 4:**
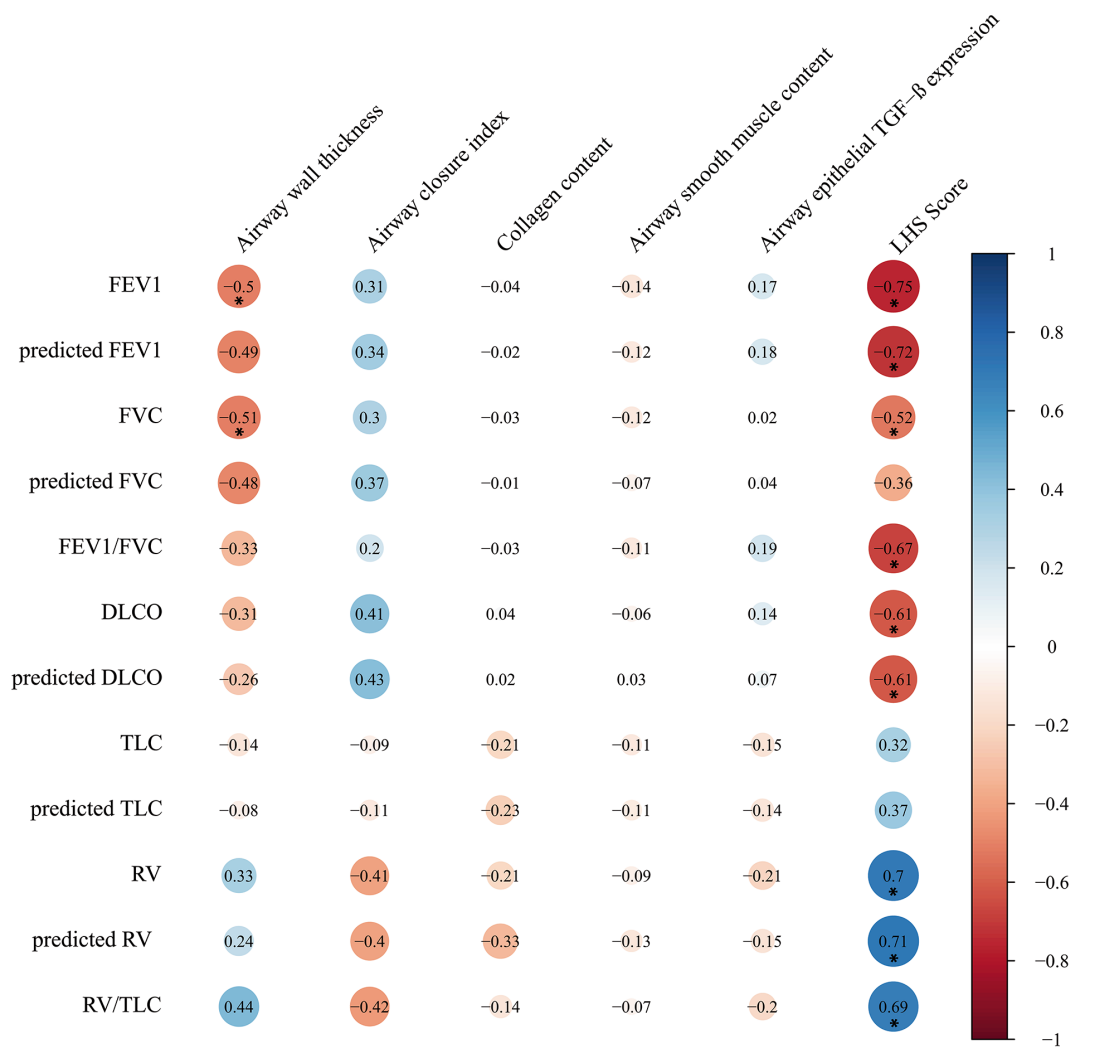
Correlation plot showing the correlation coefficients between the functional and morphological parameters in the LAM group. The correlation coefficients are color-coded from deep red (-1) to deep blue (+ 1). Predicted values are expressed as a percentage of the predicted. * indicates statistically significant correlations (p < 0.05). P-values are indicated in the main text.

Figure 4 shows the correlations between functional and morphological parameters in patients with LAM with a correlation coefficient ≥ 0.50. Airway thickness was negatively correlated with FEV1 (p = 0.008; 95%CI [-0.708, -0.225]) and FVC (p = 0.008; 95%CI [-0.682, -0.254]). LHS score was negatively correlated with FEV1 (p ≤ 0.0001; 95%CI [-0.854, -0.581]), predicted FEV1 (p ≤ 0.0001; 95%CI [-0.758, -0.213]), FVC (p = 0.007; 95%CI [-0.834, -0.545]), FEV1/FVC ratio (p ≤ 0.0001; 95%CI [-0.805, -0.446]), DLCO (p = 0.002; 95%CI [-0.831, -0.311]), and predicted DLCO (p = 0.001; 95%CI [-0.837, -0.305]). In addition, LHS score was also positively correlated with RV (p ≤ 0.0001; 95%CI [0.423, 0.862]), predicted RV (p ≤ 0.0001; 95%CI [0.487, 0.843]) and RV/TLC ratio (p ≤ 0.0001; 95%CI [0.435, 0.858]).

The correlations among the morphological parameters only showed an association between airway thickness and collagen content (r = 0.575; p = 0.004; 95%CI [0.273, 0.793]).

**Fig. 5:**
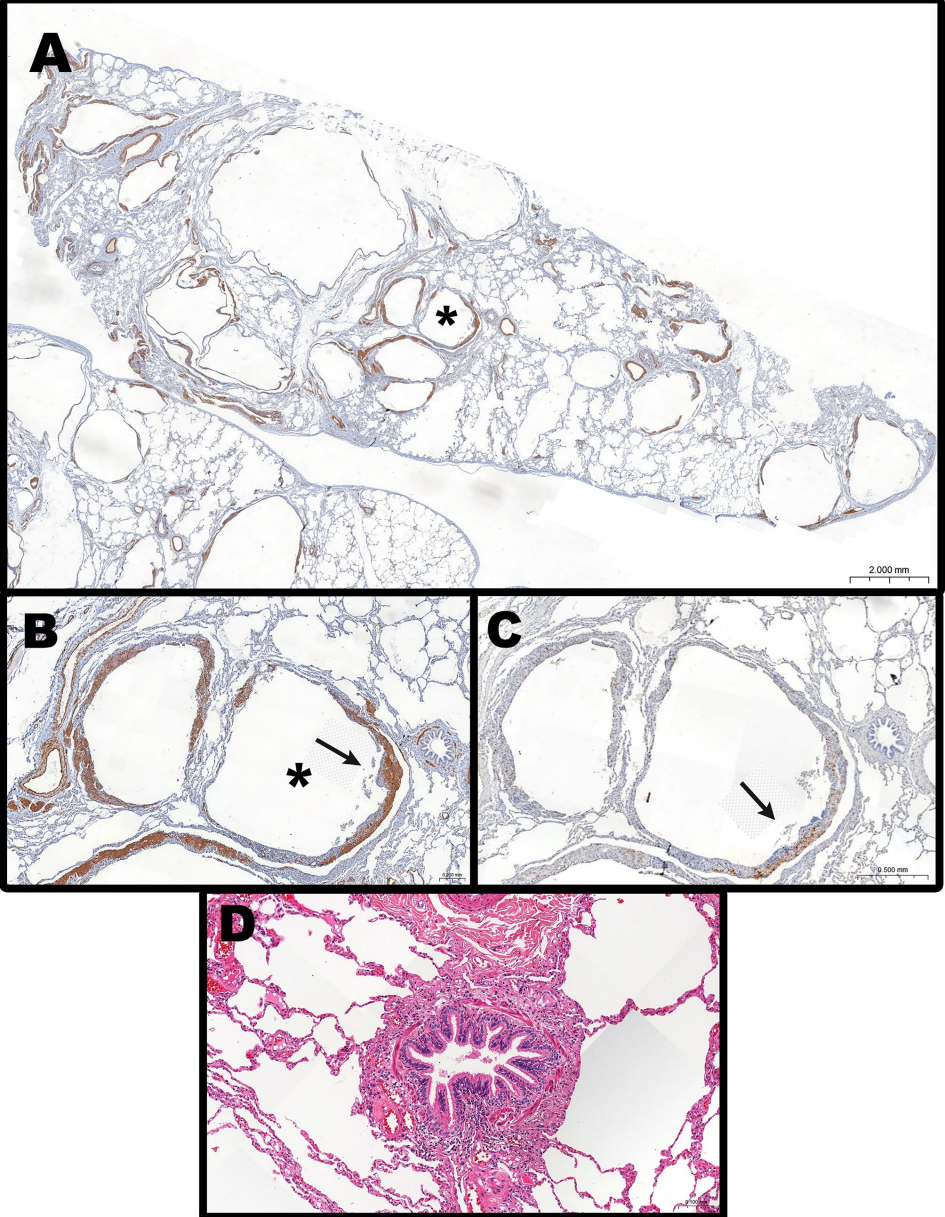
(**A**): Representative image of the cystic destruction of lung parenchyma in LAM (SMA immunostaining); (**B**): Higher magnification of peribronchiolar lung cyst (*) observed in panel A, with the typical proliferative nodule (arrow) containing SMA positive LAM cells; (**C**): The same cystic lesion as in panel B, containing HMB-45 positive LAM cells; (**D**): Photomicrograph of a small airway with mild inflammation

Figure 5 shows representative photomicrographs of LAM involvement in pulmonary tissue. Mild airway inflammation was observed in 29/39 patients (74%). Inflammatory cells consisted mainly of lymphocytes and macrophages. Several small airways did not show any inflammatory cell infiltration. Considering all patients, rare airways showed moderate inflammation containing lymphoid follicles. Airway infiltration by LAM cells was present in rare small airways of 14/39 patients (36%).

## Discussion

In this study with surgical lung biopsies and lung explants from patients with LAM at different stages of the disease, we quantitatively assessed the morphological compartments of the bronchiolar walls and their association with parameters of PFTs. Our main results are: (1) airway smooth muscle content, collagen content, the degree of small airway closure, and TGF-β epithelial expression are increased in LAM; (2) As expected, the morphological parameter of LAM severity (LHS) is associated with functional changes related to airway obstruction, air trapping and reduced DLCO; (3) small airway wall thickness is higher in patients with more severe disease and is associated with parameters of airflow obstruction and collagen content; (4) Bronchiolar inflammation was mild; infiltration of the small airways wall by LAM cells was rare.

It is well known that LAM often presents with functional abnormalities, mainly airflow obstruction, air trapping, and reduced DLCO; however, the morphological bases related to these alterations need to be better understood. Previous studies suggest that the cystic destruction of the lung tissue (which involves and incorporates altered small airways), associated with nodular proliferation of LAM cells, are the main determinants of airflow obstruction in LAM [18, 19]. Our results are in line with this concept, and further show that structural alterations of the small airways can contribute to the severity of the disease. These include partial airway closure, smooth muscle hypertrophy, and collagen deposition within the airway walls.

The hypothesis that the small airways are altered in LAM has already been partially demonstrated in a small number of studies of explants and autopsies of patients with advanced severe LAM, which demonstrated a decrease in the number of small airways (caused by cystic destruction), airway narrowing and collapse (also secondary to cystic destruction), airway inflammation, and airway infiltration by LAM cells [18, 20, 21]. The present study presents new insights into the pathophysiology of airflow obstruction in LAM, as we included patients at different stages of the disease and focused on alterations in airways with preserved structure, not involved by the cystic destruction of lung tissue. Additionally, the findings observed in our study do not seem to be associated with any other etiology. Smoking could have been a confounding factor for the morphological changes observed in the airways, but most of the patients were non-smokers. Six patients were ex-smokers, had stopped smoking at least one year before the biopsy and had a tobacco load of less than 20 pack-years. Only one patient was a smoker at the time of biopsy. For all patients, there were no relevant pathological findings suggestive of smoking. When comparing smokers and non-smokers, there were no differences in the histopathological findings (data not shown). Also, none of the patients had other relevant environmental exposures or autoimmune disorders that could be related to the airway findings. This study design allowed us to compare two groups of patients according to disease severity. Interestingly, airway thickness was the best bronchiolar morphological parameter to differentiate more and less severe patients, which was also correlated with measures of FEV1. However, airway thickness in LAM was not different from controls. As the controls came from autopsy material, it is possible that there was some degree of edema in the control airways that could have interfered with this result, which can be considered a limitation of the study.

Airway collapse due to loss of alveolar support secondary to cystic destruction has previously been suggested as a possible mechanism of obstruction in LAM [18]. We demonstrated that in addition to the possible effects of loss of alveolar attachments, there are primary structural changes in the airways, such as muscle hypertrophy and collagen deposition, which likely contributed to airway narrowing and functional abnormalities.

Unlike what has been previously reported, bronchiolitis, that is, inflammatory infiltrate in the small airways, and airway infiltration by LAM cells, were not relevant findings in our study. Taveira da Silva et al. (2001) [19] also reported the presence of bronchiolitis in LAM biopsies, which however was not a predictor of response to bronchodilators. Conversely, bronchiolar smooth muscle hypertrophy, which was an important finding in our patients, was detected in only 3 out of 74 patients evaluated by Taveira da Silva et al. (2001) [19].

Expression of TGF-β, a growth factor associated with extracellular matrix (ECM) production, has been previously described in LAM nodules. These studies suggested that the proliferation of LAM cells may be associated with altered regional expression of TGF-β1 and related ECM proteins, and postulated that TGF-β1 may promote disease progression [32]. In the present study, both the expression of TGF-β and collagen content were higher in the airways of patients with LAM when compared to controls, indicating a possible mechanism for the partial airway closure observed in our patients. TGF-β is a cytokine also associated with muscle proliferation and may be involved in the mechanism of airway smooth muscle hypertrophy. It is not possible to rule out that other mediators, not investigated in the present study and related to proliferation of LAM cells, such as growth factors (IGF-1, VEGF) and chemokines, also have a role in ASM hypertrophy in these patients.

Study limitations include the small number of patients with LAM and controls. Considering that LAM is a rare disease, this series is similar in size to other previous studies. We chose to include autopsy controls, given the high degree of tissue inflammation and changes related to smoking present in most lungs obtained in surgical procedures at our service. However, normal lungs are also uncommon in autopsies, where there may be some tissue changes related to death, such as mild edema. Due to the small number of patients with TSC-LAM, we cannot state that the etiology of LAM may have any association with the airway findings.

We conclude that LAM presents with small airway remodeling and partial airway closure, with structural alterations observed at different airway compartments, including the airway epithelium, ASM, and ECM, contributing to airflow obstruction and to disease severity. Cystic lung tissue destruction associated with nodular proliferation, assessed through LHS, seems to be the main determinant of airflow obstruction and air trapping in patients with LAM.

Data are presented as mean ± standard deviation. FEV1: forced expiratory volume in the first second, FVC: forced vital capacity, DLCO: lung diffusion capacity for carbon monoxide, TLC: total lung capacity, RV: residual volume, L: liters, % predicted: percentage of predicted value.

## Data Availability

Data are available upon request to the corresponding author.

## Abbreviations

ASM: Airway smooth muscle
BM: Basement membrane
CT: Computed tomography
CAPPesq: Comissão de Ética para Análise de Projetos de Pesquisa
DLCO: Diffusion capacity for carbon monoxide
H&E: Hematoxilin and Eosin
HMB-45: Human melanoma black-45
FEV1: Forced expiratory volume in the first second
FMUSP: Faculdade de Medicina da Universidade de Sao Paulo
FVC: Forced vital capacity
IH: Immunohistochemistry
LAM: Lymphangioleiomyomatosis
LHS: LAM Histological Score
PFT: Pulmonary function test
RV: Residual volume
sLAM: Sporadic LAM
SMA: Anti-smooth muscle alpha-actin
TLC: Total lung capacity
TSC-LAM: Tuberous Sclerosis Complex-LAM
TGF-β: Transforming growth factor beta
